# Non-invasive prenatal diagnosis of beta-thalassemia disease using digital PCR

**DOI:** 10.3389/fmed.2026.1771405

**Published:** 2026-03-30

**Authors:** Chalit Tangwerapornpong, Kuntharee Traisrisilp, Pimlak Charoenkwan, Fuanglada Tongprasert, Arunee Phusua, Kanda Fanhchaksai

**Affiliations:** 1 Division of Maternal-Fetal Medicine, Department of Obstetrics and Gynecology, Faculty of Medicine, Chiang Mai University, Chiang Mai, Thailand; 2 Division of Hematology and Oncology, Department of Pediatrics, Faculty of Medicine, Chiang Mai University, Chiang Mai, Thailand; 3 Faculty of Medicine, Thalassemia and Hematology Center, Chiang Mai University, Chiang Mai, Thailand

**Keywords:** beta-thalassemia, beta-thalassemia/Hb E disease, cell-free DNA, digital PCR, fetal fraction, NIPD, prenatal diagnosis

## Abstract

**Introduction:**

To evaluate the performance of digital polymerase chain reaction (dPCR) as a non-invasive prenatal test (NIPT) for assessing the risk of the fetus being affected by beta-thalassemia major and beta-thalassemia/Hb E disease.

**Methods:**

This cross-sectional study included 63 pregnant women from couples at risk of having a fetus with severe beta-thalassemia (homozygous beta-thalassemia or beta-thalassemia/Hb E disease), who underwent invasive prenatal diagnosis between January and September 2024. Maternal plasma cell-free DNA (cfDNA) was analyzed using dPCR to detect and quantify the paternally inherited beta-thalassemia allele (PIB) and maternally inherited beta-thalassemia (MIB) alleles. Invasive prenatal diagnosis (chorionic villus sampling, amniocentesis, or cordocentesis) served as the gold standard for fetal genotype confirmation via multiplex PCR and high-resolution melting (HRM) analysis. The mutant/normal (M/N) allele ratio was calculated, and receiver operating characteristic (ROC) analysis determined the optimal cut-off for predicting affected fetuses.

**Results:**

Among 54 couples with discordant mutations, PIB was identified in 25 cases. An MIB-M/N ratio cut-off of ≥0.919 yielded a sensitivity of 100% and a specificity of 81.2% for predicting fetal inheritance of MIB allele (AUC = 0.938). In nine couples with identical mutations, an M/N ratio of ≥1.043 predicted affected fetuses with both 100% sensitivity and specificity.

**Conclusion:**

dPCR-based NIPT accurately identifies fetuses at risk of beta-thalassemia major, potentially reducing the need for invasive procedures and associated risks. The relatively small sample size could limit the generalizability of the findings. Larger studies involving more participants from different ethnic and geographical backgrounds would provide more robust data.

## Introduction

Thalassemia is one of the most common inherited hematologic disorders, particularly prevalent in Mediterranean countries and Southeast Asia (1, 2). Carrier screening is now standard practice to perform for couples at risk of having offspring affected by severe forms of thalassemia, including homozygous alpha-thalassemia, homozygous beta-thalassemia, and compound heterozygote beta-thalassemia with hemoglobin E. To date, over 350 distinct mutations in beta-globin gene have been identified, contributing to the phenotypic heterogeneity of beta-thalassemia (3, 4). Clinical manifestations range from asymptomatic carriers (beta-thalassemia trait) to severe transfusion-dependent forms, including beta-thalassemia major (β^0^/β^0^), intermedia (β^+^/β^0^), and minor (β^+/0^/β) (5). In Thailand, the prevalence of beta-thalassemia carrier varies from 3.0% to 9.0% (6). A study conducted in northern Thailand found that the most common mutations was codon 41/42 (-TTCT) followed by codon 17 (A > T) and IVS1nt-1 (G > T) (7).

There are two primary approaches to preventing the birth of an affected child: pre-implantation genetic diagnosis (PGD) and prenatal diagnosis (PND). Couples identified as being at risk prior to conception may opt for PGD, which involves in vitro fertilization (IVF) followed by the genetic screening of embryos, allowing the transfer of only unaffected or carrier embryos into the uterus (8). This strategy avoids the need for invasive PND and eliminates the ethical and emotional challenges associated with pregnancy termination in cases where the fetus is affected. However, PGD is a costly procedure, with estimated expense ranging from 100,000 to 300,000 THB per cycle (approximately 3,000–9,000 USD), and a reported live birth rate per cycle of 30%–35% (9). Additionally, successful implementation requires close collaboration between a specialist in reproductive medicine and a well-equipped genetic laboratory, making PGD an impractical option for widespread used in current healthcare setting.

In Thailand, preconception counseling and genetic risk assessment are infrequently utilized, with only a small proportion of couples seeking evaluation prior to conception. Consequently, the primary strategy for thalassemia prevention and disease control remains prenatal screening and diagnosis. For couples identified as being at high risk of having a fetus affected by severe thalassemia, invasive prenatal diagnostic procedures, including chorionic villus sampling (CVS), amniocentesis, or cordocentesis, are performed based on gestational age to confirm the fetal genotype (2). According to data from Maharaj Nakorn Chiang Mai Hospital (Thailand) in 2024, a total of 195 pregnancies underwent invasive PND for thalassemia, indicating a relatively high number of procedures. However, these invasive procedures carry potential risks, including a 0.5%–1% risk of pregnancy loss, as well as complications such as amniotic fluid leakage, and intrauterine infection (10–12).

Cell-free DNA (cfDNA) analysis in maternal blood has gained widespread acceptance as a reliable screening method for fetal chromosomal abnormalities, particularly trisomy 21 (Down syndrome) (13–16). This technique, commonly known as non-invasive prenatal testing (NIPT), has prompted efforts to extend its application to the detection of other genetic disorders, including thalassemia. Digital polymerase chain reaction (dPCR) has emerged as a promising technology for the highly sensitive and precise detection of genetic mutations (17). Unlike the conventional PCR method, dPCR enables quantitative analysis by partitioning the DNA sample into numerous individual reactions, thereby allowing for the detection of rare mutant alleles even in the presence of an overwhelming excess of wild-type alleles (18, 19). If successfully adapted for thalassemia testing, dPCR-based NIPT could offer a viable alternative for detecting homozygous beta-thalassemia, and compound heterozygote beta-thalassemia with hemoglobin E as early as the first trimester, thereby reducing reliance on invasive diagnosis procedures and minimizing associated maternal-fetal risks.

This study aims to evaluate the effectiveness of dPCR-based NIPT for predicting whether the fetuses are affected by homozygous beta-thalassemia or beta-thalassemia/Hb E disease, based on the analysis of cfDNA in maternal blood samples.

## Materials and methods

### Ethical approval

This study was conducted at Maharaj Nakorn Chiang Mai Hospital, Thailand, between January and September 2024. Ethical approval was obtained from the Research Ethics Committee of the Faculty of Medicine Chiang Mai University (Research ID: OBG-2566-0460).

Pregnant women between 11 and 23^+5^ weeks of gestation, who were at risk of carrying a fetus affected by severe beta-thalassemia (β^0^/β^0^) or compound heterozygote beta-thalassemia-Hb E disease (β^0^/β*^E^*), were enrolled. Risk status was identified through the standard national thalassemia screening program in Thailand. Only couples harboring one or more of the four common HBB mutations, including codon 41/42 (-TTCT), codon 17 (A > T), IVS1nt-1 (G > T), and Hb E mutation, codon 26 (G > A) were enrolled into the study. All participants and their partners provided written informed consent prior to sample collection. A total of 12 mL of maternal blood was collected from each participant. The pregnant women underwent invasive PND, including CVS, amniocentesis, or cordocentesis, depending on gestational age and patient preference. The fetal HBB genotype was determined using multiplex PCR combined with high-resolution melting (HRM) analysis, and the results were recorded for further analysis.

### Plasma cell-free DNA extraction

A total of 12 mL of maternal peripheral blood was collected in two 6 mL EDTA tubes and stored at 4 °C until processing, which occurred within 2 h after collection. Samples were initially centrifuged at 3,000 rpm for 10 min to separate plasma. The supernatant was then carefully transferred to new tubes and subjected to a second centrifuged at 14,000 rpm for 10 min at 4 °C to remove residual cellular debris. The resulting plasma was stored at −20 °C until further processing. Cell-free DNA (cfDNA) was extracted from the plasma using the QIAamp circulating Nucleic Acid Kit (QIAGEN, Hilden, Germany) according to the manufacturer’s instruction.

### Primer and probe sequence

The primers and probes used to detect the four common mutations of HBB; codon 41/42 (-TTCT), codon 17 (A > T), IVS1nt1 (G > T), and codon 26 (G > A) were as described previously (20, 21).

### dPCR assay

The dPCR assay was designed to detect and quantify both paternally inherited beta-thalassemia allele (PIB) and maternally inherited beta-thalassemia allele (MIB) from cfDNA in maternal plasma. The quantification was performed using the QIAcuity Digital PCR System (Qiagen, Hilden, Germany). The assay included the reaction setup, thermal cycling conditions, data acquisition and analysis. Each 40 μL reaction mixture contained 1X QIAcuity Probe PCR master mix, 0.4 μM of each wild-type and mutant alleles, TaqMan probes specific to HBB mutations including codon 17 (A > T), codon 41/42 (-TTCT), codon 26 (G > A), IVS1nt1 (G > T). A fixed volume of 5 μL of extracted genomic DNA was added to each dPCR reaction. DNA extraction was performed using a commercial kit, which yields DNA concentrations typically ranging from 5 to 16 ng/μL and DNA purity (OD260/280 ratio) ranging from 1.8 to 2.2, according to the manufacturer (Qiagen, Hilden, Germany). The reaction mixtures were loaded into a QIAcuity Nanoplate 26k, 24-well format (QIAGEN, Hilden, Germany), and the nanoplate was transferred to the QIAcuity instrument for amplification and analysis. The dPCR thermal cycling protocol consisted of polymerase activation at 95 °C for 2 min, then 40 cycles of denaturation at 95 °C for 15 s and annealing-extension at 60 °C for 1 min. All samples were processed using the same standardized protocol to ensure consistency across reactions.

Each family’s test utilized 12 wells of the nanoplate, organized as follows: five wells with multiplex primers and probes to detect MIB-mutant allele (MIB-M) and MIB wild-type allele (MIB-N); one well with a positive heterozygous MIB control; one no-template control (NTC); three wells with multiplex primers and probes to detect PIB-mutant allele (PIB-M) and PIB wild-type allele (PIB-N); one well with a positive heterozygous PIB control; and one no-template control (NTC). Results were reported as the numbers of positive, negative and void partitions, corresponding to presence or absence of target DNA molecules.

### Fetal fraction and sex determination analysis

The fetal fraction and sex determination were analyzed and calculated by iSAFE*™* NIPT Gender and Fetal Fraction Determination Assay Kit (ATILA BioSystems, Sunnyvale, California, United States) according to the manufacturer’s instruction.

### Analysis of dPCR results

The analysis focused on evaluating the presence or absence of PIB-M and quantifying the ratio of MIB-M to MIB-N from dPCR data. For PIB-M analysis, the total PIB-M count from triplicated analysis was compared between the fetuses who inherited versus those who did not inherit the paternal mutation. The presence of PIB-M indicated the fetus had inherited the parental mutant allele, suggesting either a beta-thalassemia carrier status (if only the paternal mutation was inherited) or compound heterozygous beta-thalassemia disease (if both paternal and maternal mutations were inherited). The absence of PIB-M indicated the fetus did not inherit the paternal mutation, and the possible fetal diagnoses were either normal (no HBB mutations) or carrier of the maternally inherited mutation only.

For MIB analysis, the total MIB-M/MIB-N ratio was calculated using data from five replicate wells and compared between the fetuses who inherited versus those who did not inherit the maternal mutation. An evaluated MIB-M/MIB-N ratio was indicative of fetal inheritance of the maternal mutation, while a lower ratio suggested non-inheritance.

In the couples who carried the same HBB mutations, the M/N ratio was used to differentiate affected fetuses from carriers and unaffected ones, by comparing ratio between the fetuses who inherited versus those who did not inherit the mutation.

The optimal cutoff value for the MIB-M/N ratio to distinguish hereditary maternal mutations was determined through subsequent ROC analysis.

It is important to note that the analysis was based on the numbers of positive PIB and MIB partitions obtained from the nanoplate of dPCR. Absolute DNA copy number was not calculated.

### Statistical analysis

The statistical analysis was performed using SPSS version 27.0 (IBM Corporation, New York, United States). Categorical variables were analyzed using Chi-squared test or Fisher’s exact test, as appropriate. Continuous variables were analyzed using student *t*-test, Kruskal–Wallis test and Mann-Whitney U test, depending on data distribution. To determine the optima; cut-off values for distinguishing between affected and unaffected fetuses, receiver operating characteristic (ROC) curve analysis was performed. A *p*-value < 0.05 was considered statistically significant.

## Results

A total of 63 pregnant couples at risk of having a fetus affected by homozygous beta-thalassemia or compound heterozygote beta-thalassemia-Hb E disease were enrolled. Based on whether both partners carried identical or different HBB mutations, all participants were divided into two categories: those with different mutations (different PIB-M and MIB-M in Table 1) were classified as the compound heterozygous β-thalassemia risk group (*n* = 54); whereas those with identical mutations (identical PIB-M and MIB-M in Table 1) were classified as the homozygous β-thalassemia risk group (*n* = 9).

**TABLE 1:** The type of hemoglobin subunit beta gene (HBB) mutations in the couples at risk.

Different paternal and maternal mutations (*n* = 54)		HBB mutation (*N* = 63)
Paternal mutation (PIB-M)	Maternal mutation (MIB-M)	*N* (%)
Codon 41/42 (-TTCT)	Codon 26 (G > A)	12 (19.0)
Codon 26 (G > A)	Codon 17 (A > T)	11 (17.5)
Codon 26 (G > A)	Codon 41/42 (-TTCT)	11 (17.5)
Codon 17 (A > T)	Codon 26 (G > A)	8 (12.7)
Codon 26 (G > A)	Ivs1nt-1 (G > T)	5 (7.9)
Codon 41/42 (-TTCT)	IVS1nt-1 (G > T)	3 (4.7)
Codon 41/42 (-TTCT)	Codon 17 (A > T)	2 (3.2)
Codon 17 (A > T)	Codon 41/42 (-TTCT)	1 (1.6)
IVS1nt-1 (G > T)	Codon 26 (G > A)	1 (1.6)
**Identical paternal and maternal mutations (*n* = 9)**		
**Paternal mutation (PIB-M)**	**Maternal mutation (MIB-M)**	
Codon 17 (A > T)	Codon 17 (A > T)	5 (7.9)
Codon 41/42 (-TTCT)	Codon 41/42 (-TTCT)	3 (4.8)
IVS1nt-1 (G > T)	IVS1nt-1 (G > T)	1 (1.6)

Among the 63 risk couples enrolled, the most common HBB mutation from different PIB-M and MIB-M was codon 41/42 (-TTCT) with codon 26 (G > A) (19.0%), followed by codon 26 (G > A) with codon 17 (A > T) (17.5%), and codon 26 (G > A) with codon 41/42 (-TTCT) (17.5%) (Table 1). All pregnant women underwent invasive prenatal diagnosis, with amniocentesis being the most common (58.7%), and the average gestational age was 18.28 weeks. The highest average fetal fraction was observed in those who underwent cordocentesis (10.03%) (Table 2). The gestational ages at the time of blood sampling and invasive prenatal diagnosis, along with the mean fetal fraction (FF) are presented in Table 2. The mean FF was 10.03% (9.83–10.23) in maternal plasma who underwent cordocentesis, 9.37% (6.19–11.86) for CVS, and 7.89% (6.26–9.52) for amniocentesis, respectively.

**TABLE 2:** Gestational age and fetal fraction of pregnant women who underwent prenatal diagnosis.

PND (*n* = 63)	Number (%)	Gestational age (weeks)	Fetal fraction (%)
Amniocentesis	37 (58.7)	18.28 (17.57–19.00)	7.89 (6.26–9.52)
CVS*	24 (38.1)	12.71 (12.28–13.43)	9.37 (6.19–11.86)
Cordocentesis	2 (3.2)	21.78 (20.57–23.00)	10.03 (9.83–10.23)

*CVS, chorionic villus sampling.

**FIGURE 1:**
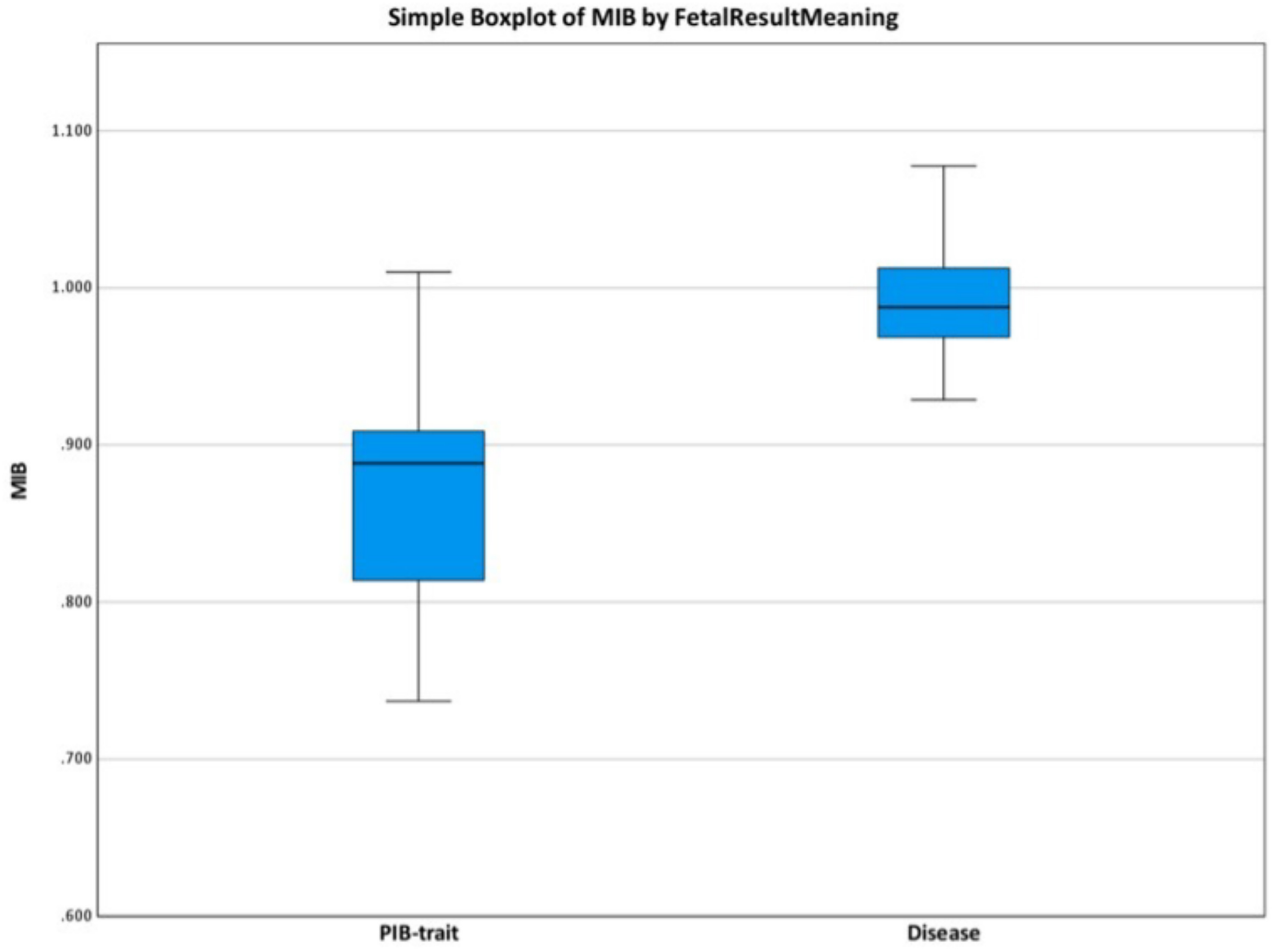
Boxplot comparison of maternally inherited beta-thalassemia-mutant/normal (MIB-M/N) ratio in paternally inherited beta-thalassemia allele (PIB) positive samples, the PIB-trait fetus and affected fetuses in different parental and maternal mutation.

**FIGURE 2:**
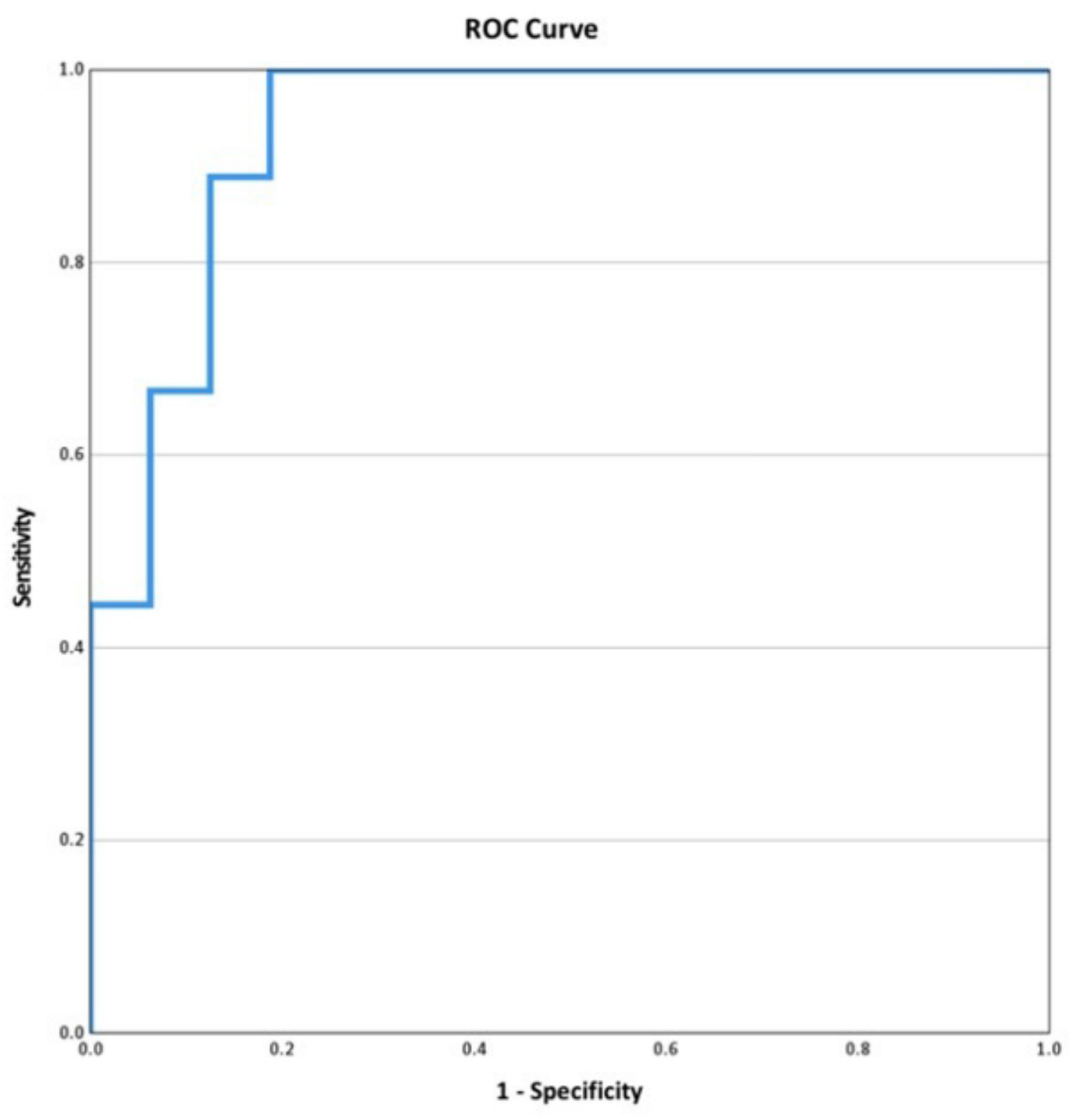
The receiver operating characteristic (ROC) curve shows the performance of MIB-M/MIB-N ratio to predict affected fetuses in PIB-M positive samples (AUC 0.938; 95% CI 0.847–1.028; *p* < 0.01).

Among the 54 couples with different paternal and maternal mutations, the paternally inherited beta-thalassemia mutation (PIB-M) was presented in 25 samples (46%). In these 25 cases, the MIB-M/N ratio was calculated to differentiate the affected fetuses from those with the paternal inherited beta-thalassemia trait. The MIB-M/N ratio was significantly higher in affected fetuses (0.994 ± 0.045) compared to those with the PIB-trait (0.870 ± 0.076) (*p* < 0.01) (Figure 1). The ROC analysis of MIB-M/N ratio was conducted with area under the curve (AUC) of 0.938 (95% CI, 0.847–1.028) (Figure 2). A threshold MIB-M/N ratio of 0.919 demonstrated 100% sensitivity and 81.2% specificity in differentiating affected fetuses from PIB-trait carriers.

**FIGURE 3:**
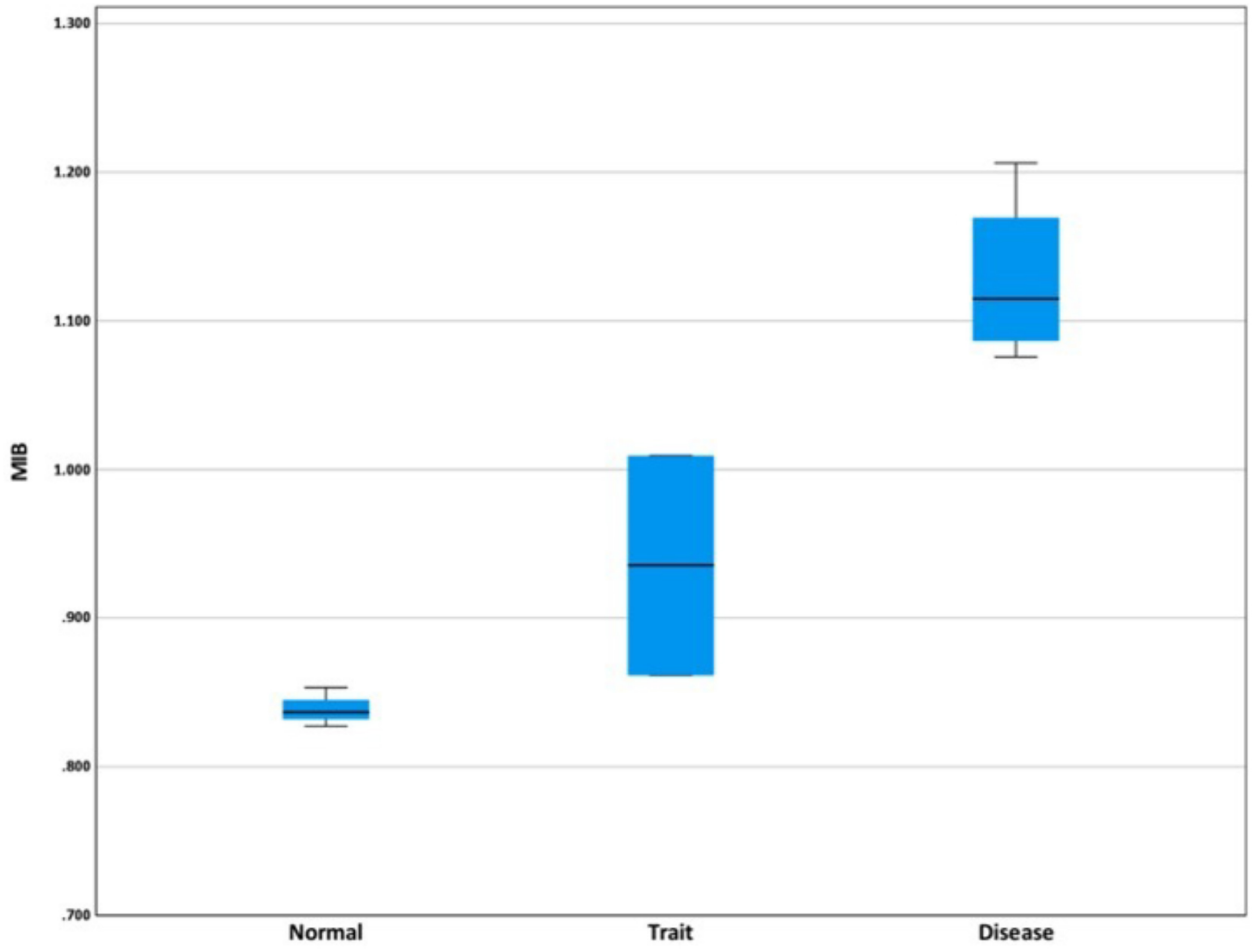
Boxplot comparison of mutant/normal (M/N) ratio of normal, trait, and affected fetuses in same paternal and maternal mutation.

**FIGURE 4:**
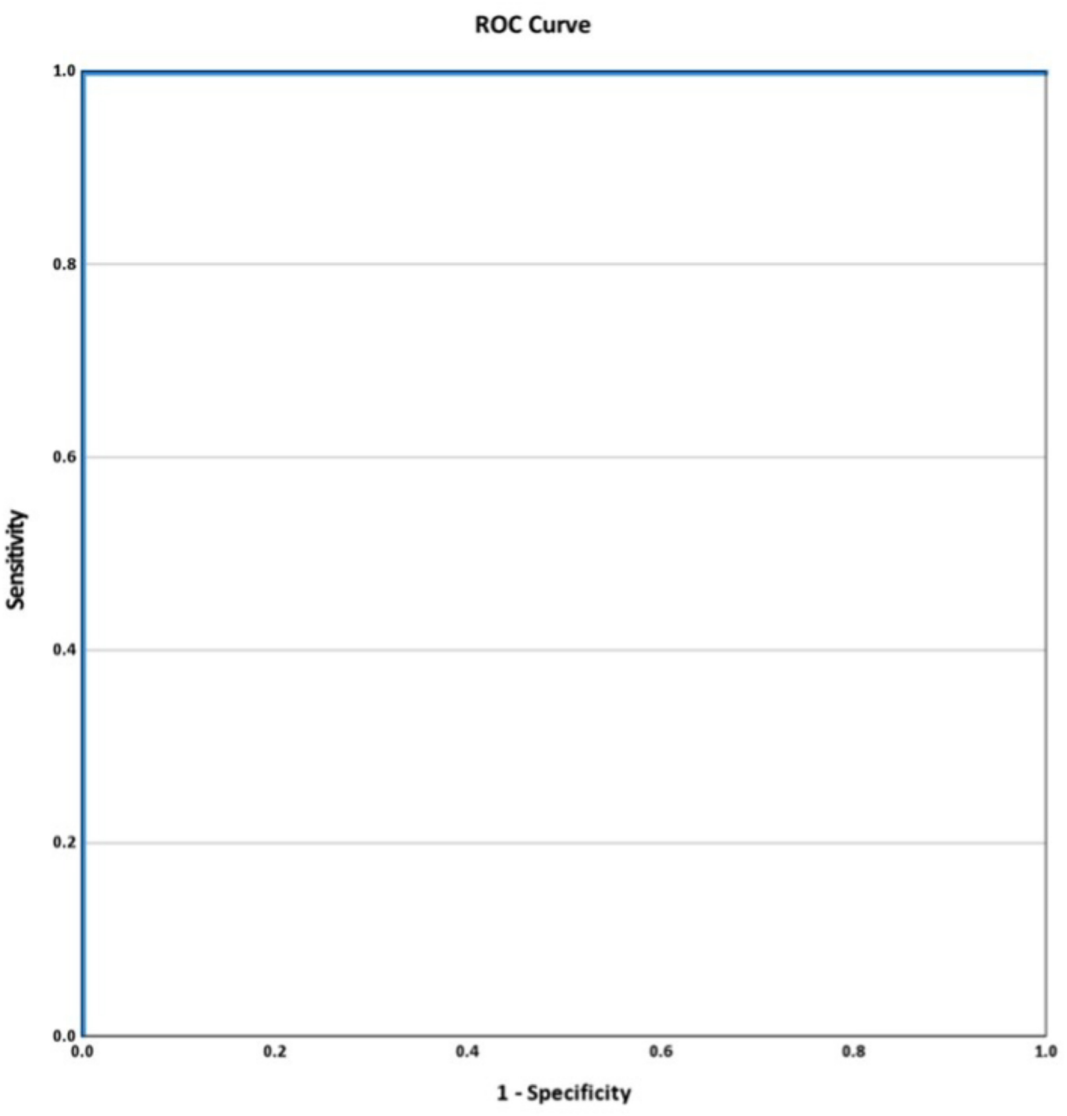
The receiver operating characteristic (ROC) curve shows the performance of mutant/normal (M/N) ratio to predict fetuses in same paternal and maternal mutation of nine couples (AUC 1.000, *p* < 0.01).

In nine couples harboring identical paternal and maternal mutations, the M/N ratios was used to differentiate between normal, carrier, and homozygous beta-thalassemia fetuses. The mean M/N ratios for each group were 0.839 ± 0.013, 0.935 ± 0.105, and 1.128 ± 0.057, respectively (Figure 3). The mean M/N ratio in homozygous beta-thalassemia fetuses was significantly higher than in carriers and normal fetuses (*p* = 0.03). The ROC analysis demonstrated an area under the curve (AUC) of 1.000. An M/N ratio exceeding 1.043 exhibited 100% sensitivity and 100% specificity in distinguishing affected fetuses from unaffected fetuses (Figure 4).

## Discussion

This study aimed to evaluate the effectiveness of NIPT using dPCR for detection of beta-thalassemia in at risk fetuses. A total of 63 pregnant couples, with gestational ages ranging from 11 to 23^+5^ weeks, were enrolled. The dPCR-based NIPT results were compared to those obtained through standard invasive prenatal diagnostic procedures including CVS, amniocentesis, and cordocentesis. The results demonstrate that dPCR offers high diagnostic accuracy and holds strong potential as a reliable, non-invasive alternative for the prenatal diagnosis of beta-thalassemia, particularly in cases of homozygous beta-thalassemia and compound heterozygote beta-thalassemia-Hb E disease.

**FIGURE 5:**
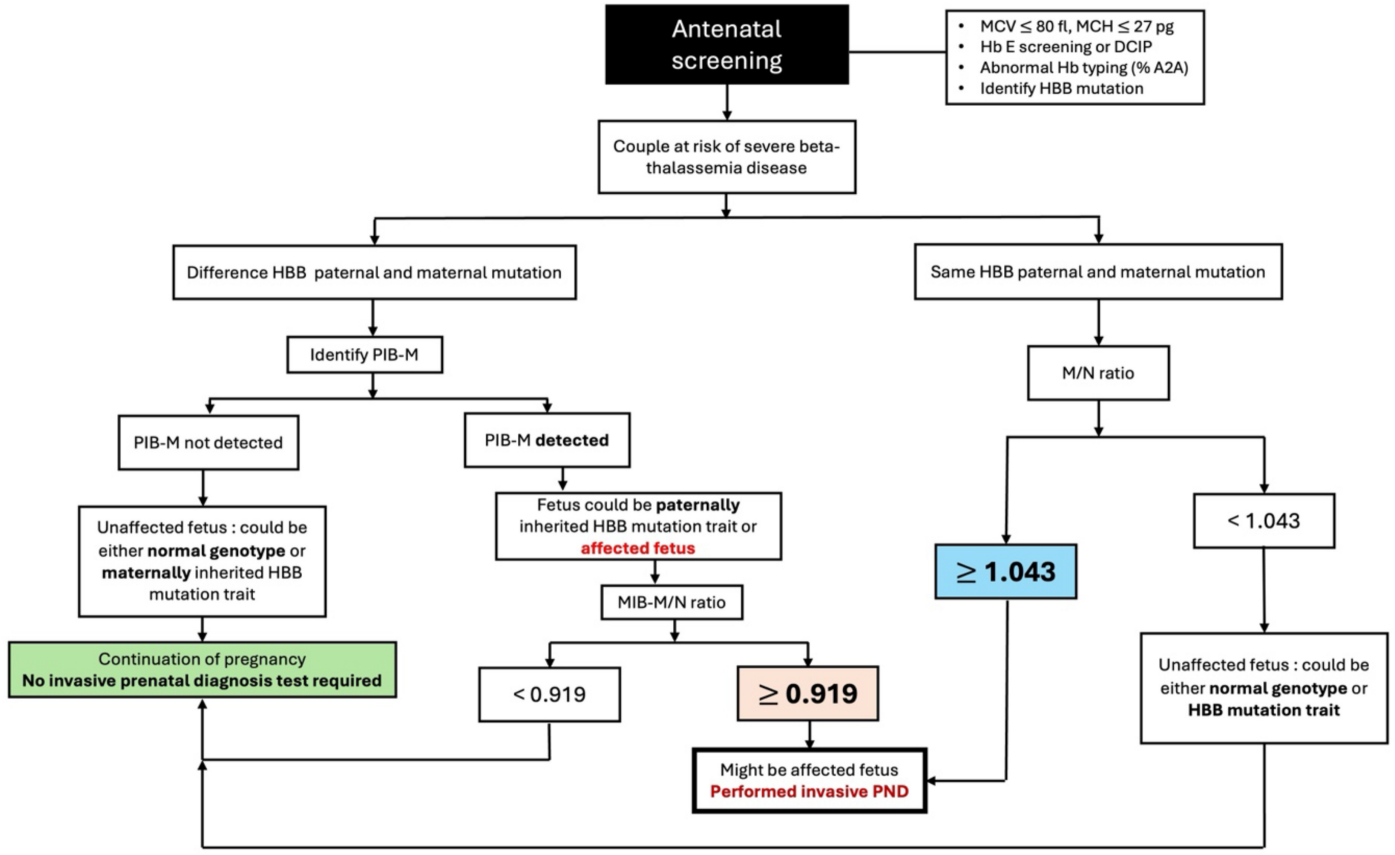
Proposed algorithm for screening fetuses at risk of severe beta-thalassemia using digital polymerase chain reaction (dPCR) as a screening method.

The proposed clinical application of dPCR for non-invasive prenatal diagnosis is illustrated in Figure 5. In couples carrying different paternal and maternal HBB mutations, the dPCR workflow begin with the detection of PIB-M. If PIB-M is not detected, it means that the fetus can either carry a normal genotype or carry only the maternal inherited beta-thalassemia trait. In such cases, the pregnancy can safely continue without the need for invasive prenatal diagnosis. This approach could potentially eliminate the need for invasive testing in up to 50% of cases, thereby minimizing procedural risks. If PIB-M is detected, the next step is to determine the MIB-M/N ratio to determine whether the fetus also inherited the maternal mutation. In theory, affected fetuses would contribute additional mutant alleles into maternal plasma, resulting in an elevated MIB-M/N ratio, ideally approaching or exceeding 1.0. However, previous studies have shown that technical artifacts such as damaged droplets, non-specific amplification, and irregular droplet size, can lead to false- negative partitions (20–22). Therefore, a fetal diagnosis should not be based solely on an M/N ratio ≥ 1.0. Instead, an optimal cut-off must be determined to prioritize sensitivity, ensuring affected fetuses are not missed. This study demonstrates that an MIB-M/N ratio of ≥0.919 predicts affected fetuses, achieving a 100% sensitivity and 81.2% specificity, offering a clinically actionable threshold. By following this approach, invasive prenatal diagnosis can be avoided in approximately two-thirds of at-risk pregnancies, thereby reducing the associated risk of fetal loss. Our result highlights the sensitivity of dPCR for detecting genetic mutations, even in the presence of a large excess of normal alleles, supporting its used as a powerful tool in non-invasive prenatal diagnostics.

In the subset of couples with identical paternal and maternal HBB mutations, this study also demonstrated the clinical utility of the M/N ratio in distinguishing affected fetuses from unaffected ones. A threshold M/N ratio of ≥1.043 provides 100% sensitivity and 100% specificity in differentiating affected fetuses from unaffected ones. If the M/N ratio does not meet this threshold, invasive testing can be safely avoided, thereby reducing procedural risks.

In addition, this study also assessed the fetal fraction (FF) to enhance the reliability of dPCR-based NIPT results, as FF serves as a critical quality control parameter in non-invasive prenatal testing (23). While the minimum FF required for optimal NIPT performance varies depending on the specific assay, most studies suggest a threshold of greater than 4% (15, 24). A higher FF increases confidence in the results interpretation (24). In this study, all participants had an FF exceeding 4%, meeting quality criteria for valid analysis. Interestingly, no positive correlation was observed between FF and gestational age, which contrasts with finding from a meta-analysis by Mousavi et al. (25), that reported an increasing FF with gestation. However, a negative correlation between FF and maternal BMI was observed, consistent with previous literature (26).

This high diagnostic accuracy of dPCR, combined with the ability to specifically detect beta-thalassemia mutations, position it as a valuable tool for early prenatal screening. This approach has the potential to significantly reduce the need for invasive diagnostic procedures such as amniocentesis and CVS, which are associated with procedural risks including miscarriage (10–12).

The findings of this study underscore the promising potential of dPCR as a non-invasive method for prenatal diagnosis of beta-thalassemia. The results highlight the high sensitivity, even in the presence of a large excess of wild-type alleles, demonstrating its suitability for detecting rare fetal mutations within maternal plasma. By offering a highly sensitive and specific alternative to invasive procedures, dPCR-based NIPT could significantly enhance prenatal care, particularly in populations at high risk for thalassemia. Moreover, the ability to perform this screening as early as the first trimester, allowing for timely clinical decision-making and genetic counseling, ultimately improving outcomes for affected families.

Nevertheless, several limitations of this study should be considered. First, the research was conducted in a specific population, which may limit the generalizability of the finding. Further studies across diverse ethic and geographic populations are necessary to evaluate the broader applicability and diagnostic performance of dPCR. fv, while the study focused on detecting common beta-thalassemia mutations, the effectiveness of dPCR to detect rare or novel mutations remains unexplored and warrants future studies. Third, small sample size could limit the generalizability of the findings, particularly when considering diverse populations or rare mutations. Larger cohort studies involving participants with a wider genetic backgrounds and mutation types would provide more robust and externally valid data, especially for detection of less frequent mutations or variants. Fourth, all participants in this study had a fetal fraction greater than 4%, a threshold generally considered sufficient for reliable non-invasive prenatal testing. However, the minimum FF threshold at which dPCR retains diagnostic accuracy remain unknown. Further studies should address this by evaluating performance at lower FF to better define the assay’s limitations and potential in early gestation or in individuals with high maternal BMI, which may lower FF.

## Conclusion

Digital PCR (dPCR) has emerged as a highly precise and innovative approach for the non-invasive prenatal diagnosis of beta-thalassemia, demonstrating excellent diagnostic performance and promising clinical utility. This advanced technology offers a viable alternative to conventional invasive procedures, with the potential to improve clinical outcomes while reducing the risks associated with prenatal diagnosis interventions.

## Data Availability

The datasets analyzed for this study are not publicly available due to patient confidentiality and ethical considerations but are available from the corresponding author upon reasonable request.
